# Low mood, not anxiety, connected with micro facial expression recognition

**DOI:** 10.1038/s41598-025-26921-1

**Published:** 2025-11-28

**Authors:** Kasia Wezowski, Ian S. Penton-Voak

**Affiliations:** 1 School of Psychological Science, University of Bristol, Bristol, UK; 2 National Institute for Health Research Bristol Biomedical Research Centre, University Hospitals Bristol NHS Foundation Trust, Bristol, UK

**Keywords:** Health care, Psychology, Psychology

## Abstract

Previous research has demonstrated that facial expression recognition, an invaluable social skill, may be impaired amongst people suffering from anxiety. Research surrounding this relationship is equivocal and little attention has been given to the effects of anxiety on the recognition of micro expressions. Thus, the present study investigated this relationship. Based on previous research, we expected that participants with high trait anxiety will show (a) poorer overall micro expression recognition and (b) better angry face recognition. 401 participants completed measures of trait and state anxiety, depression, micro facial expression recognition and indicated demographic information. The results of the study supported neither of the two hypotheses. Combined with previous findings, these results indicate that trait anxiety does not have a robust effect on either general emotion recognition or anger recognition. Previous positive findings may potentially be a consequence of unaccounted effects of low mood or age. On the other hand, the results did show effects of low mood on improved overall micro expression recognition scores and sad face recognition, and a bias towards recognizing neutral faces as sad. These findings may be attributed to biases arising from the effects of mood congruence.

## Introduction

Facial expressions are a basic, relatively universal way of expressing emotions^1^. Their accurate interpretation is a crucial skill in social interactions^2,3^, which not all people are equally good at^4^. The ability may be reduced in those suffering from a variety of mental disorders, including depression and anxiety^5^. Reduced social aptitude is a characteristic of various mental health issues^6^, including anxiety, a debilitating disease affecting a large number of people, with the Global Burden of Disease Study 2019 estimating 45.82 million incident cases of anxiety disorders worldwide^7^.

The literature on the relationship between various forms of anxiety and facial expression recognition is far from unequivocal. The literature on emotion recognition in anxiety is not uniform. Some work reports broad disruptions in the recognition of facial expression^5,8–12^. Other studies find no overall impairment, even in clinical or sizeable non-clinical samples, indicating that anxiety does not invariably produce a general decoding deficit^10,13^, while some research even demonstrates better overall recognition of emotions^14^. A large and more consistent cluster of findings points to emotion-specific effects, most often a heightened sensitivity to threat cues (anger and/or fear): anxious participants (especially socially anxious or clinically anxious groups) detect anger or fear at lower intensities or earlier in morph sequences^14–22^. A smaller but notable set of studies reports interpretation biases, with ambiguous or neutral faces being more likely to be labeled as angry or threatening^20,21,23–25^. Other studies make the picture more complex by showing anxiety-linked enhanced recognition for some emotions or in some tasks (e.g., low-trait sensitivity to happiness or better recognition of certain emotions in higher-anxiety participants), underlining that effects vary in direction and are task-dependent^14,26,27^.

Several methodological and sample factors (outlined in detail in Table 1) help explain these mixed outcomes. First, we can consider the anxiety construct that is measured: experimentally induced state anxiety tends to produce immediate reductions in accuracy and/or increases in bias^10,11^, social anxiety measures and social anxiety clinical samples more reliably predict threat-specific sensitivity and labeling biases toward anger^17,20,21,23–25,28,29^, whereas trait anxiety, generalized anxiety disorders, or mixed clinical presentations sometimes show effects, but not in always^8,13,16,30,31^. Second, sample type and clinical status matter: many of the clearest group differences emerge in clinical samples^8,9,12,17^, while non-clinical samples sometimes do^10,14,19^, but sometimes also do not find effects^13,32^. Third, stimulus and task properties are crucial: morphs and graded-intensity stimuli, brief or masked presentations, and tasks that measure detection thresholds or early recognition frequently reveal sensitivity at low intensities^15,18,24,26,28,30^, whereas long exposure or unlimited viewing and forced-choice formats can attenuate group differences^13,14,33^.

**Table 1:** Previous research investigating the relation between anxiety and facial expression recognition.

Study	Sample	Anxiety measurement	Facial expression ability measurement	Findings
Richards et al., 2002^26^	Non-clinical, *N* = 60 + 74	STAI + SPAI (groups)	Morphed images of faces between pairs of happiness, surprise, fear, sadness, disgust, and anger	Low-trait group sensitive for happiness.
Philippot & Douilliez, 2004^33^	Clinical (SP, other anxious disorders, HC), *N* = 21 + 20 + 39	CA	Happiness, anger, sadness, disgust, and fear demonstrated at different intensities	No differences amongst groups.
Joorman & Gotlib, 2006^15^	Clinical (MDD, SP, HC), *N* = 23 + 27 + 26	CA + STAI (groups)	Anger, disgust, fear, happiness, neutral, sadness, and surprise, intensities varied between 0 and 100%, presented for 500ms	SP participants more sensitive to anger at lower intensity.
Surcinelli et al., 2006^16^	Non-clinical, *N* = 39	STAI (groups)	Identification of seven expressions: anger, sadness, happiness, fear, surprise, disgust, neutral	Participants with high trait anxiety recognized fear expressions significantly better; no group differences on recognition accuracy for other emotions.
Mohlman et al., 2007^17^	Clinical (gSAD), *N* = 26 + 26 HC	CA + SPS + STAI (groups)	Line drawings of neutral, happy, sad, and angry expressions, with different intensities.	gSAD participants more sensitive towards anger, especially low-intensity
Cooper et al., 2008^13^	Non-clinical, *N* = 109	STAI	Anger, disgust, fear, happiness, neutral, sadness, and surprise, images presented on screen until answer is given	No anxiety-related differences in emotion perception were observed across all seven emotions.
Demenescu et al., 2010^5^	Clinical (various), *N* = 355 from 5 studies	Various (groups)	Meta-analysis of previous research on anxiety and facial expression recognition	There is a detrimental effect of anxiety on emotion recognition.
Bell et al., 2011^23^	Clinical (gSP), *N* = 30 + 27 HC	CA (groups)	Anger, disgust, fear, happiness, neutral, sadness, and surprise, intensities varied between 0 and 100%, presented for 500ms	No differences between the groups on emotion recognition; SP group was more likely to misclassify facial expressions as angry.
Palm et al., 2011^31^	Clinical (GAD), *N* = 15 + 16 HC	HAMA interview, STAI (groups)	Faces with expressions of anger, disgust, fear, happiness, sadness and surprise were presented at 10 different intensities, for 500ms	GAD group less accurate detection of sad facial expressions compared with control participants.
Button et al., 2013^24^	Non-clinical, *N* = 102	BFNE (extreme groups)	Five emotions (anger, disgust, fear, happiness, and sadness) expressed at 15 intensities (9–65%),	Social anxiety was not associated with overall decoding accuracy but was associated with a response bias. Highly socially anxious were more likely to recognize emotions at lower intensities, but also did so wrongly more often.
Doty et al., 2013^18^	Non-clinical, *N* = 21	STAI	Short presentation of fear, happy, or neutral (33ms) followed by a neutral mask, answer - fear or not fear	Trait, but not state anxiety correlated with fear detection
Gebhardt & Mitte, 2014^19^	Non-clinical, *N* = 116 + 76	MAS	Happiness and anger presented in lower and higher intensities	Higher anxiety connected to interpreting milder anger as stronger.
Yoon et al., 2014^20^	Non-clinical, *N* = 89	SIAS	Happiness and anger presented in lower and higher intensities (morphed with neutral faces)	Higher social anxiety associated with sensitivity to mild anger and tendencies to label facial expressions as angry. No relation with mildly happy faces.
Jusyte & Schönenberg, 2014^32^	Clinical (gSAD), *N* = 32 + 32 HC	SIAS + LSAS + SPS (groups)	Ambiguous faces “between” three emotions (angry, happy, fearful)	No effects of social anxiety on emotion detection
Berg et al., 2016^30^	Clinical (MDD), *N* = 14 with + 14 without anxiety	HAM-D anxiety-somatization score (groups)	The Emotional Expression Multimorph Task - faces morphing from neutral towards an emotion, earlier recognition = better recognition	Anxiously depressed less sensitive to happiness and sadness at lower intensities. Severity of anxiety correlated with greater intensity of emotion required to detect sad faces. Sensitivity to anger and fear was not observed in anxious depression.
Torro-Alves et al., 2016^28^	Non-clinical, *N* = 43	SPIN (groups)	Happiness, anger, fear, sadness, neutral, different intensities, static vs. dynamic	Social anxiety more sensitive to low intensity static anger, no differences on dynamic emotions.
Attwood et al., 2017^11^	Non-clinical, *N* = 21 + 43 + 1994	STAI	Six-alternative forced choice (6AFC) task (face morphs)	Experimental studies – state anxiety connected to reduced emotion recognition accuracy and increased interpretation bias (a tendency to perceive anger over happiness). Observational study – higher state anxiety associated with poorer emotion recognition, negative effects of trait anxiety nullified when controlling for state anxiety.
Tseng et al., 2017^12^	Clinical (SAD), *N* = 31 + 31 controls	CA (groups)	Facial emotion recognition task, forced choice (happy, sad, angry, fearful)	SAD patients were significantly slower and less accurate at recognizing facial expressions, mainly due to slower and less accurate recognition of faces showing fear. Sad faces recognition was reduced amongst more depressed participants within the SAD cohort.
Ferrer da Rosa et al., 2017^14^	Non-clinical, *N* = 150	STAI	Faces of happiness, sadness, fear, and anger varying in emotional intensity from the neutral to the expressive face in steps of 25%, faces presented for 2s.	Better recognition of happiness, anger, and fear in more anxious participants
Gutiérrez-García & Calvo, 2017^21^	Non-clinical, *N* = 48	STAI + SPS (groups)	Dynamic face morphs (from neutral to emotion), lasting 1s in total	Social anxiety associated with enhanced detection of anger and disgust at low intensity levels, relative to non-anxious controls. Social anxiety related to interpreting emotionally “neutral” faces as angry.
Kang et al., 2019^22^	Non-clinical, *N* = 42	K-BAI (Korean version BAI)	Face morphs of different emotion intensities (angry, happy, sad)	Anxiety correlated with sensitivity for angry faces but not for sad or happy faces.
Dyer et al., 2021^10^	Non-clinical, *N* = 48	STAI	Six-alternative forced choice (6AFC) task (face morphs)	Induced state anxiety reduced global emotion recognition accuracy, no relationship between trait anxiety and global emotion recognition accuracy. Greater interpretation bias towards anger during heightened state anxiety among individuals with high trait anxiety.
Leung et al., 2022^29^	Non-clinical, *N* = 188	SIAS	Ambiguous faces “between” three emotions (angry, disgusted, happy)	Social anxiety connected to poorer sensitivity in recognizing threatening faces. No effect of interpretation bias.
Fujihara et al., 2023^27^	Non-clinical, *N* = 138	STAI + BFNE	Six emotions (happy, sad, fear, anger, disgust, surprise) shown at 20%, 40%, and 100% intensity	Higher trait anxiety connected to predicted faster reaction times to fearful faces at 40% intensity; higher social anxiety predicted misidentifying happy faces as disgust at 40% intensity; higher state anxiety predicted fewer misidentifications of happy as sad at 20% intensity and fewer fixations on the eye region for low-intensity sad or fearful faces.
Lacombe et al., 2023^9^	Clinical (SAD), *N* = 788 across 16 studies	CA (groups)	Emotion expression recognition tasks using validated face-databases	SAD participants show significantly poorer overall accuracy in recognizing emotional expressions (especially happiness) compared to controls.
Williams et al., 2023^27^	Clinical (CHR), *N* = 62 + 52 HC	SIAS (groups)	Penn Emotion Recognition-40 Test - happy, angry, sad, fearful, or neutral expressions, multiple choice	Within CHR participants, social anxiety related to anger detection bias.
Ren et al., 2025^8^	Clinical (GAD), *n* = 60 + 60 HC	GAD-7, HAMA (groups)	Seven facial expressions (neutral, happy, sad, angry, surprised, fearful and disgusted) presented for 3s	Significant differences between GAD vs. control for neutral, angry and fearful expressions.

BAI = Beck Anxiety Inventory; BFNE = Brief Fear of Negative Evaluation; CA = clinical assessment; CHR = Clinical High Risk for Psychosis; GAD = generalized anxiety disorder; GAD-7 = generalized anxiety disorder 7-item inventory; gSAD = generalized social anxiety disorder; HAMA = Hamilton Anxiety Rating Scale; HC = healthy controls; LSAS = Liebowitz Social Anxiety Scale; MAS = Manifest Anxiety Scale; MDD = major depressive disorder; SAD = social anxiety disorder; SIAS = Social Interaction Anxiety Scale; SP = social phobia; SPIN = Social Phobia Inventory; SPS = Social Phobia Scale; STAI = State-Trait Anxiety Inventory.

Where N is represented by multiple numbers, these either refer to multiple studies or to the number of experimental (diagnosed) and healthy control subjects.

The effects of anxiety on emotion expression recognition can be well understood from an evolutionary perspective. Anxiety is part of the body’s stress response system which prepares an organism for encountering threats^34^. Accordingly, enhanced sensitivity to others’ emotional expressions, especially anger, can be adaptive, since detecting such emotions early allows faster reactions and potentially aids in avoiding danger^35^. Therefore, in line with the aforementioned findings about anxious people being able to detect these types of faces at lower intensities^13,19,22,28,30,32^, it can be expected that detecting micro expressions, a more subtle and fast type of emotion expression than macro expressions^36^, will also be altered amongst more anxious individuals. In particular, anxious individuals may show enhanced recognition of anger but reduced recognition of other emotions. The impairment in recognizing non-threat emotions could stem both from (a) an attentional bias toward threat and (b) cognitive costs associated with anxiety that reduce overall processing capacity^37^.

Anxiety is not the only mental health disorder that has been linked to differences in facial expression recognition. Depression, the fourth leading contributor to the global disease burden^38^, has also been shown to have a relationship with facial expression recognition. Similarly to anxiety, it has been shown to have three main types of effects. One is an overall effect on reduced capability of emotion recognition^5,39–41^, which is usually attributed to a deficit in cognitive processing associated with depression. The second effect is one on easier recognition of specific emotions, which has most commonly been recognized for sadness^5,42^, but also for other emotions, such as disgust^43^, fear^41,44^, and surprise^45^. Finally, people with higher depression scores tend to have a bias in expression recognition towards sadness – seeing happy faces as neutral, and neutral faces as sad^41,43,45^.

An important and less researched aspect of facial expressions is the domain of micro expressions. Micro expressions are short (< 0.5 s), spontaneous presentations of the seven basic emotions^36,46–48^. Even if a person is trying to hide their true emotions, micro expressions still appear^49^, making their detection advantageous in social situations. In the recent years, a novel method of measuring micro expressions recognition, the Micro Expression Training Videos (METV) has been developed^50–53^. This method utilizes short videos of emotions instead of static images like Ekman’s Micro Expression Training Tools^54^, which improves its ecological validity, since people normally do see facial expression in a dynamic manner. Higher METV scores reflect a higher ability in recognizing micro expressions. The importance of micro expression, as measured by the METV, is also reflected in the fact that it has been demonstrated that people with low mood have altered micro expression recognition^52^, suggesting a link with mental health. In spite of this, there is no previous research on the relationship between anxiety and micro expressions.

Here we focus on two of the most salient findings in the literature. The first is that there might be a relationship between anxiety and overall facial emotion recognition^5,8–12^ with some research indicating a lack of an effect as well^13,24,28^. The studies which found the effect found it either for one of the subscales (trait or state) of the state-trait anxiety inventory^10,11^ or for diagnosed anxiety disorders^8,12^. As the scope of the study does not allow for clinical estimation and diagnostics, trait anxiety was seen as the most adequate independent variable to use. Therefore, the first hypothesis of the study was:

### H1

There will be a negative relationship between trait anxiety and global METV scores.

A second common finding in the literature is that people suffering from higher levels of anxiety tend to be more sensitive towards angry face recognition^14,17,20–22,25^. While different measures of social and trait anxiety were used in previous research, we have opted to keep trait anxiety as the main independent variable for the sake of consistency and simplicity. Thus, the second research hypothesis was:

### H2

There will be a positive relationship between trait anxiety and angry face METV scores.

Although there are other findings in the literature indicating other effects of anxiety on facial recognition, we abstained from creating study hypotheses for each of the potential effects. Instead, the abovementioned two are the main hypotheses of the study, while other potential relationships that may arise will be determined in exploratory analyses of the data. As studies have also indicated effects of state anxiety^10,11^ and depression^5,40,41^, we have also measured these constructs to be used as control variables and in exploratory analyses.

Additionally, we attempted to replicate the findings of a previous study which investigated the relationship between low mood and micro expression recognition^52^. By testing the same hypotheses as those explored in the previous study, the present one will test the robustness of its findings and bring additional clarity to the relationships between micro expression recognition and mental health. These hypotheses are considered exploratory for the current study, as they were not planned for prior to data collection, and the sample size calculation was not performed based on them. These are the additional hypotheses, meant to replicate the mentioned previous study^52^.

1. Low mood will be connected to lower overall METV scores.
2. Low mood will be connected to higher sad face score.
3. Low mood will be connected to mistaking happy faces for neutral ones.
4. Low mood will be connected to mistaking neutral faces for sad ones.

## Results

### Sample characteristics

The initial sample consisted of 448 participants. 47 participants (9.5% of overall sample) were removed as they had either a missing global METV score, or reported issues with the testing. No further extreme outliers were identified. Thus, the final sample consisted of 401 participants (Table 2). Since 1 respondent did not report their gender, analyses adjusted for gender as a control variable pertain to smaller samples of 400 respondents (200 female). Participants had an average age of 43.63 (range 18–88, SD = 15.09). Neither of the two independent variables (trait subscale of STAI, BDI-II) showed normal distribution (Shapiro-Wilk *p* < .001), which is why the continuous variables were transformed into dichotomous groups. The distribution of scores and the descriptive statistics across the two study groups regarding trait anxiety, as well as the two study groups regarding low mood, may be found in the supplementary materials (Tables S1 to S4, Figures S1 and S2). While there are some differences between groups on demographic variables, these differences will be controlled for in the adjusted models presented in the following sections. This was done to prevent possible confounding effects of these variables, as they have been found to influence facial expression recognition or cognitive performance in general in previous research^55,56^.

**Table 2:** Descriptive statistics of key study variables.

Variable	Mean	Std. dev.	Min	Max
METV first correct answers	6.72	2.53	0	14
Angry face first correct answers	0.21	0.21	0	1
Trait anxiety	43.25	4.57	25	55
State anxiety	44.94	4.92	30	62
Depression symptoms (BDI-II)	12.98	12.11	0	49

*Note. n* = 401.

### Association between trait anxiety and METV performance

Prior to interpreting the results, it was determined that no assumptions of regression analyses were broken. The Durbin-Watson statistic was 1.880, indicating no violation of autocorrelation of residuals, VIF values were close to one, and residuals demonstrated a normal distribution upon visual inspection of Q-Q plots. No evidence was found for a negative relationship between trait anxiety and recognising human emotions, in the unadjusted or adjusted (age, gender, education, and low mood) regression analyses. Similarly, no evidence was found for an association between overall anxiety or state anxiety and global METV scores. In the adjusted analyses, very strong evidence was found for an association between age and METV performance of participants (*B* = -0.031, CI = -0.049 – -0.014, *p* < .001), indicating that younger participants display higher levels of competence in recognising human emotions from facial expressions. There was also a marginal level of evidence for the impact of low mood on METV results, with participants in the low mood group showing higher scores. The final model explained 4% of METV first answer variance (adj. *R*^2^ = 0.04), making the observed post-hoc power of the study 0.92. Full details of the models are reported in Table 3.

**Table 3:** Regression models predicting METV first correct answers.

Predictors	Unadjusted		Model 1		Model 2	
	*b* [95% CI]	*p*	*b* [95% CI]	*p*	*b* [95% CI]	*p*
High trait anxiety group	– 0.116	0.651	– 0.065	0.797	– 0.025	0.923
	[– 0.618, 0.386]		[– 0.463, 0.432]		[– 0.525, 0.476]	
Age			– 0.036	< 0.001	– 0.031	< 0.001
			[– 0.052, – 0.020]		[– 0.049, – 0.014]	
Gender			– 0.165	0.507	– 0.147	0.556
			[– 0.656, 0.325]		[– 0.638, 0.343]	
Education			0.035	0.782	0.039	0.757
			[– 0.213, 0.283]		[– 0.209, 0.287]	
Low mood group					0.368	0.173
					[– 0.162, 0.899]	

*Note. n* = 400. Ordinary Least Squares regression. Model 1: Adjusted for age, gender, and education level. Model 2: Adjusted for depression symptoms (low mood/control), age, gender, and education level.

### Association between trait anxiety and angry face METV scores

Prior to interpreting the results, it was determined that no assumptions of regression analyses were broken. The Durbin-Watson statistic was 1.912, indicating no violation of autocorrelation of residuals, VIF values were close to one, and residuals demonstrated a normal distribution upon visual inspection of Q-Q plots. Neither unadjusted nor adjusted (age, gender, education, low mood) regression analyses have provided any evidence in support of the second hypothesis of an association between trait anxiety and angry face METV scores. In the adjusted analyses, marginal evidence was found for an association between age and angry face METV performance of participants (*B* = -0.001, CI = -0.003–0.000, *p* = .065). Full details of the models are reported in Table 4.

**Table 4:** Regression models predicting angry faces first correct answers.

Predictors	Unadjusted		Model 1		Model 2	
	*b* [95% CI]	*p*	*b* [95% CI]	*p*	*b* [95% CI]	*p*
High trait anxiety group	– 0.005	0.827	– 0.004	0.835	– 0.005	0.804
	[– 0.047, 0.037]		[– 0.047, 0.038]		[– 0.048, 0.037]	
Age			– 0.001	0.065	– 0.001	0.066
			[– 0.003, 0.000]		[– 0.003, 0.00]	
Gender			– 0.025	0.237	– 0.026	0.230
			[– 0.067, 0.017]		[– 0.067, 0.016]	
Education			0.002	0.879	0.002	0.886
			[– 0.019, 0.023]		[– 0.020, 0.023]	
Low mood group					– 0.008	0.723
					[– 0.053, 0.037]	

*Note. n* = 400. Ordinary Least Squares regression. Model 1: Adjusted for age, gender, and education level. Model 2: Adjusted for depression symptoms (low mood/control), age, gender, and education level.

### Post-hoc tests on continuous trait anxiety as a predictor

In order to determine if the null findings of the main hypotheses were a consequence of the main predictor (trait anxiety) being used as a binary variable, instead of the numeric variable it was recorded as, post-hoc analyses were performed with its original form. Age, gender, and education were used as control factor in the same way as in the previous analyses, and the original BDI-II score was used as well. The analyses showed no evidence for the effect of trait anxiety on METV scores, both before, *t* = – 0.24, *p* = .811, and after, *t* = -0.05, *p* = .961, adjusting for demographics and BDI (full models in Table S5). Similarly, there was no effect of anxiety on anger recognition both before, *t* = 0.14, *p* = .888, and after, *t* = 0.15, *p* = .881, adjusting for demographics and BDI (full models in Table S6).

### Associations between low mood and METV test performance

Exploratory analyses were undertaken in order to investigate the effects of low mood on micro expression recognition. We tested whether there were effects on overall METV scores, sad face recognition, and biases of perceiving neutral faces as sad and happy faces as neutral. Since 40 individual regression coefficients were determined in this post-hoc analysis, a Bonferroni-corrected alpha value of 0.00125 (0.05/40) was considered strong evidence of an effect, while values between 0.05 (the traditional threshold) and 0.00125 (the Bonferroni-corrected one, which is considered conservative^57^ were considered weak evidence. The details of these analyses may be found in supplementary materials. A linear regression indicated weak evidence that low mood was predictor of overall METV scores (*B* = 0.729, *p* = .004) when not controlling for other variables (full models in Table S7). After introducing the demographic variables and trait anxiety, the evidence of relationship was lost (*B* = 0.371, *p* = .166). Another linear regression (full models in Table S8) indicated strong evidence that low mood predicted sad face recognition before (*B* = 0.316, *p* < .001), but weak evidence after (*B* = 0.073, *p* = .009) controlling for other variables. A binary logistic regression showed no evidence for effects of low mood on mistaking happy faces as neutral, either before or after controlling for other variables (full models in Table S9). Finally, there was marginal evidence of an effect of low mood on mistaking neutral faces for sad ones after (but not before) controlling for demographics and trait anxiety (Exp (*B*) = 0.291, *p* = .062). Based on the Exp(*B*) coefficient, participants in the low mood group were between three and four times as likely to have mistaken the neutral face as a sad one (Full models in Table S10). However, the effect in the present study was not strong enough to be considered significant. We also tested for an interaction effect of low mood and anxiety on all four dependent variables using a series of ANCOVAs and found no significant interaction effects (all *p* > .05).

## Discussion

The present study examined the effects of anxiety on micro facial expression recognition. The main study hypotheses were that trait anxiety would have a negative effect on micro facial expression recognition and a positive effect on the recognition of angry faces. The study results did not support either of the two hypotheses. Further analyses were conducted in order to attempt replication of a previous study^52^ by assessing the effects of low mood on micro expression recognition.

The finding that there was no effect of anxiety on overall micro expression recognition is in line with many previous studies^13,24,28^. Other research did find such a relationship^10–12^. The differences can be accounted for by the fact that these effects may have been a consequence of either induced anxiety^10,11^ or an interplay between low mood and anxiety^12^. A meta-analysis^5^ also indicated a general effect of anxiety on emotional recognition. However, the studies which their assessment is based off are mostly those showing an effect on bias, and not on general emotion recognition^15,17,58–60^. Some of them also showed effects limited to only certain emotions^16^ or ones that can be better explained by depression^61^. In short, although Demenescu et al.^5^ state that anxiety leads to a “moderate impairment of facial emotion recognition in adults” (p. 3), this impairment may only be an effect of bias in interpretation, and not a deficit in facial emotion expression recognition per se.

Based on our findings and in agreement with those of previous research^13,24,28^, it seems that there is no robust association between anxiety and the overall recognition of facial expressions. The present study’s results additionally support this notion by showing that the relationship is absent in micro expression recognition as well. As we have also demonstrated an effect of low mood on general micro expression recognition, it may be that the previous studies which reported effects of anxiety simply failed to account for their participants’ depression symptoms. Additional investigations may be needed in order to determine if there is any effect of anxiety on facial expression recognition that can be isolated from the common comorbidity with depression and its symptoms.

The second study hypothesis, which postulated that angry face recognition would be improved amongst participants with higher trait anxiety, was not supported by the study findings. Participants with lower and higher levels of trait anxiety were equally sensitive to micro expressions of anger, which is in line with some^10,30^, but out of line with other previous research^14,17,20–22,25^. This is likely a consequence of the fact that all but two^14,22^ of the cited studies which found this result utilized measures or diagnoses of social anxiety disorders. Some of the previous studies which used measures of general anxiety also found no effects on higher sensitivity towards anger^10^. Another relevant aspect of the studies is that all but one of them^25^ showed the strongest effects of anxiety on low-intensity anger. This can be interpreted as a consequence of increased vigilance to threat^20^ which is notable with low-intensity depictions of anger, but not with those of higher intensity, as the emotions are too clear. In sum, while social anxiety may have an effect on increasing vigilance towards angry faces, general anxiety does not seem to. This is likely because general anxiety is focused on aspects of life that do not involve social interactions, and is, thus, not as connected to the hypervigilance to threat from others.

In addition to examining the effects of anxiety on emotion recognition, the present study also investigated depressive symptoms to replicate previous findings^52^. Consequently, these results will be primarily discussed in relation to that study. The present study’s results showed weak evidence of an effect of low mood on METV scores in the unadjusted model. When adjusted for demographic characteristics and trait anxiety, the evidence of the relationship was lost, with age emerging as the strongest predictor, indicating that the effects of age and low mood are connected. The positive effect of low mood on overall METV scores is a consequence of the increased accuracy in recognizing sad faces, which is proven by the fact that the association is lost once the analysis is adjusted for sadness recognition. These findings contrast with those of the replicated study^52^, which reported a negative effect of low mood on micro expression recognition, as measured by the METV. The authors attributed this decline to cognitive impairment associated with low mood. However, that study found no evidence of an association between low mood and improved sadness recognition. The discrepancy between the studies may be due to differences in average depression scores, which were somewhat lower in the present study. This suggests that in the replicated study, cognitive decline associated with depression may have overshadowed any heightened sensitivity to sadness, whereas the opposite pattern emerged here. However, this interpretation remains speculative and would require further research explicitly accounting for cognitive impairment. Age has been found to be an important, negative predictor of micro expression recognition. Previous research agrees with this finding^56,62,63^ and has indicated that the likely reason for this is that the volume of the “social brain”, mainly located in frontal and temporal lobes, is reduced with age^64^.

Our study also found some indicators of interpretation and attention biases in participants with higher BDI-II scores. There was strong evidence for a higher sensitivity for sad faces and marginal evidence for a propensity to misjudge neutral faces as sad. There was no bias in interpreting happy faces as neutral. These findings are in line with previous research on sad face sensitivity^65^ and biases towards recognizing neutral faces as sad^41,43,45,52^. The previous study^52^ which attempted to find these increased sensitivities found only the bias in interpreting neutral faces as sad, but not an overall increased sensitivity towards sadness, and the difference might be due to the larger sample size in the present study. Both of the findings may be interpreted through mood congruence, which affects the way a person perceives, interprets, or remembers things, by painting them with their current emotional state^66^. More specifically, mood-congruence theory proposes that a person’s current affective state selectively tunes both attention and memory toward mood-consistent information, thereby shaping perception at multiple stages of processing^66^. In the context of micro-expression recognition, a depressed mood may heighten early attentional capture by sad cues (attentional bias), leading individuals to orient more quickly and sustain gaze on sadness-related features of a face^15^. Aside from attentional bias, this phenomenon may also be caused by interpretation bias. More depressed individuals are more likely to interpret ambiguous stimuli as negative, which includes neutral facial expressions^66^.

The present study finds a stark difference in the effects of anxiety and low mood. While they are both mental health disorders that are thought to similarly affect emotion perception, the mechanism by which this occurs is different. Negative attentional bias and the coloured perception of neutral or happy facial expressions can occur differently in individuals who suffer from anxiety and those who suffer from depression. This may explain the difference in results of the study between the two disorders. A number of studies^67–69^ find that anxiety often manifests in heightened sensitivity to threat cues, leading to a bias towards interpreting ambiguous situations as threatening. Conversely, a number of studies find depression is characterized by deficits in emotional processing^39–41^, such as reduced reactivity to positive stimuli and increased attentional focus on negative information. Thus, the mechanisms by which they affect emotion recognition vary. While the necessary contextual factors for the effect of low mood on emotion recognition may have been present in the study, the same cannot be said about anxiety. The heightened sensitivity required in the case of anxiety may not have been elicited by the images used in the METV test, while no such arousal of threat response is necessary in the case of depression.

### Limitations and strengths

The present study had several limitations. One is that we were not able to collect a sample from a clinically diagnosed population with anxiety disorders. Instead, we used online convenience sampling and a survey to measure the variance in anxiety amongst this unknown population. Thus, as the maximum trait anxiety in the sample was 55, and the theoretical maximum of the trait STAI sub-scale is 80, it is clear that we have not had people with very high anxiety in the sample. Hence, this lack of variance in trait anxiety may have contributed to the lack of evidence of the relationships. Yet, while limiting our findings to a non-clinical population, the study sample was still sufficiently large and heterogenous to capture some existing effects. This is also confirmed by comparing the STAI scores and the results to previous research which used the same instrument. While there are previous studies which had a wider range of STAI scores and found some significant effects^11,16^, there are also those which also had a wider range and did not find such effects^13^. Furthermore, there is also literature on research which had an overall lower^18^ or similar^10^ score range to those presented here, and it also shows a mix of positive and null findings. Therefore, we do not believe that the limited range of STAI scores was the reason behind the null effects.

Furthermore, since the STAI scores were not normally distributed, we used a median-split in order to avoid violating the assumptions of the general linear model. This loses some variability in the independent variable, but is also in line with the tradition in the literature, which is very commonly group-based, regardless if these groups are based off clinical diagnoses or splitting a sample based on anxiety scores (Table 1).

Another limitation is that we have not included a measure of social anxiety, which may be necessary in order to fully understand the relationship between anxiety and facial expression recognition. Yet, there is certainly collinearity between trait anxiety and social anxiety, and the present study focused on the former. Thus, the investigation of the effect of social anxiety on micro expression recognition should be conducted in a future study. Furthermore, the hypotheses about low mood that were tested were not set forward prior to the study, but were added after the data collection. While they are still based on previous research, they were not pre-planned, which introduces some potential bias. A further limitation is that the METV task includes only the seven classic Ekman^49^ emotions (anger, sadness, fear, surprise, disgust, contempt, happiness) and a neutral face, and therefore does not capture the recently documented “anxiety” facial expression^70,71^. If individuals with high trait anxiety are especially sensitive to this specific signal, our design may have underestimated their ability to detect anxiety-related cues. Future studies could address this by incorporating validated stimuli of the anxiety expression.

A final limitation concerns the design of the METV test itself. In particular, the test presents participants with an unequal number of stimuli across emotions. For example, only a single happy face and a single neutral face were shown, which may have limited variability in responses to these categories and reduced the ability to detect effects. This imbalance could also have biased the sensitivity of emotion recognition across different facial expressions. At the same time, happiness is conveyed primarily by one key movement – the upward lift of the lip corners – so including multiple examples might create a learning effect. For other emotions, which involve a wider range of facial muscle patterns, participants again saw only one instance of each movement. Providing several examples in those cases could likewise introduce a different kind of bias. In the end, a trade-off was inevitable, but it remains important for future research to examine whether the present findings hold when alternative stimulus sets are used.

The study also had several strengths. One was that we utilized a sufficiently large sample, which was projected from previous research on the power of expected results. Thus, the absence of support for our hypotheses may not be attributed to an insufficiently large sample. The sample was also heterogenous in terms of age, gender and education. Another strength is that we utilized STAI and BDI-II, questionnaires with well-supported validity and reliability from previous research. We also utilized METV, a relatively new instrument that has shown reliability and validity as well, and is further validated by the findings of the present study which indicate that it shows very similar relationships as those found in previous research. Furthermore, a part of the study was a replication of previous findings, which is very important for the development of psychological sciences^72^. Finally, we included demographic and other control variables in all regression models, which prevented potential false positive findings, thus improving the validity of the present results.

## Conclusions

The present study had the main objective of investigating the effects of trait anxiety on general micro facial expression recognition, as well as on the recognition of angry faces. Controlling for demographic variables and low mood, we found no such effects. Hence, the main novel finding of the present study is the lack of association between micro expression processing, as measured by METV, and anxiety. A secondary objective of the study was to replicate previous findings on the relationship between low mood and METV performance. In this regard, we found effects of low mood on overall METV performance, sad face sensitivity, and a bias towards recognizing sadness in neutral faces, which is in only partially in line with the findings of the replicated study, demonstrating a necessity for additional research. Our findings indicate that trait anxiety may not be relevant for micro facial expression recognition. However, low mood likely has an important effect on attention and interpretation biases, and these effects are not yet fully understood, given the fact that the findings of the present study are only partially aligned with those of the one we replicated. Hence, additional research should be conducted in order to understand the details of the relationship between facial expression recognition and various issues with mental health. Such studies will help us work on various programs that could improve the social functioning and quality of life of persons struggling with these debilitating conditions.

## Methods

The study was pre-registered on OSF: https://osf.io/mqud7/. Ethics approval was obtained from the Faculty of Science Research Ethics Committee at the University of Bristol (Approval Code: 2023-15983-17913). The study was conducted according to the revised Declaration of Helsinki (2013) and the 1996 ICH Guidelines for Good Clinical Practice E6(R2). All methods were performed in accordance with the relevant guidelines and regulations. Data collection started on 26/10/2023, and ended on 10/12/2023. The investigator explained the nature, purpose and risks of the study to the participants in an online information sheet before they consented to participate in the study by clicking a button. Participants were informed that they were free to withdraw at any time by simply closing the web page. Thus, all participants were provided informed consent during the study. All data was anonymized before analysis.

### Study design

This study used an observational, cross-sectional design. The primary measures in the study were the participants’ overall scores on the METV micro expression recognition test, the score on the anger faces, and the groups based on the scores of the trait anxiety sub-scale of the State-Trait Anxiety Inventory (STAI). The main independent variable of the study was the anxiety group created by splitting the sample into two equally-sized parts, based on the participants’ trait anxiety score. Thus, the trait anxiety group was a categorical, nominal variable with two levels (low anxiety/high anxiety).

The first dependent variable of the study was the participants’ overall METV score, which was calculated as the proportion of correct answers given to the test on the first try. Thus, it is a numeric variable measured on the ratio level. The theoretical minimal and maximal scores are 0 and 1. The second dependent variable was the participants’ score on angry faces, which is also calculated as the proportion of correct answers given on the first try. It is also a numeric variable measured on the ratio level, with the theoretical score ranging from 0 to 1.

### Participants and recruitment

Participant recruitment was done through Prolific. Initial screening done by the platform allowed us to exclude participants who currently used psychotropic medication, as research demonstrated that it has an effect on emotion recognition^73^. We also used the pre-screening to create a diverse sample in terms of their self-reported anxiety. Due to the limited nature of the way in which it was measured on the platform (a simple yes/no answer), this was not taken into account when creating participant groups in the study, but instead their scores on the STAI were used for that purpose. Furthermore, as these pre-screening questionnaires were completed an unknown time before recruitment into the study, medication use was checked in the study survey. Only participants over 18 were recruited for the study, they had to be fluent in English, and they were reimbursed £4.

#### Sample size determination

It has been shown in a meta-study that the average effect size for the effect of anxiety on facial expression recognition is *d* = – 0.35^5^. Using the G*Power software, we determined that the sample size needed to achieve this effect size (with the assumption that this effect size will be similar for micro expression recognition) at 0.95 power is 428. Therefore, the aimed sample size was 450 (225 per group), so that there would be a sufficient number of participants after outliers and non-completers are removed from the sample.

It should be noted that the sample size is based on studies which utilized macro facial expressions as stimuli. This was the only realistic source of effect size information because the literature on micro expression recognition (and its relationship with mental health) is sparse. It is possible that the true effect sizes of the effects investigated in the present study are smaller, which will be determined in the analyses. Also, post hoc power calculations will be done in order to determine if the used sample size was sufficient to observe any effects in the sample.

#### Withdrawal of participants

Participants were informed that they were able to withdraw from the study at any time by leaving the study webpage. Participants who opted out before completing the survey did not receive a reimbursement.

### Measures and materials

#### Micro expressions training videos (METV)

The METV^51^ measured micro expression recognition ability based on the detection of micro expressions on videos. It is based on the Facial Action Coding System rules (Ekman & Friesen, 1978). There were 20 videos, each showing a male or female white person presenting a single micro expression, for 0.5 s or shorter. Since each emotion can be demonstrated by a different number of micro expressions^74,75^, the emotions were not represented in equal numbers of stimuli – there were 4 micro expressions of anger, 4 of sadness, 3 of fear, 3 of surprise, 2 of disgust, 2 of contempt, 1 of happiness, and 1 neutral face. Once the emotion is presented, the participants answer by selecting one of the eight possible answers (7 emotions + neutral). Completing the test took about 10 min. While additional answers (up to 3) are allowed to participants if they make a mistake on the first try, only the proportion of correct answers on the first try was used as the dependent variable in the present study.

The reliability of the METV has so far not been determined. This is due to the fact that the test was primarily developed for education purposes, and as such – gives feedback on whether an answer was correct or not and allows the participant to answer multiple times until a correct answer is given. Hence, test-retest reliability could not be determined, because it is expected to have a learning effect, as has been demonstrated in a previous study^53^. The test had also demonstrated face and expert validity, which has been communicated by participants and experts in micro-expression recognition. Finally, it showed excellent construct validity through expected correlations with other psychological constructs, such as correlating positively with emotional intelligence^76^ and negatively with depressive symptomatology^52,53^.

#### The State-Trait anxiety inventory (STAI)

The State-Trait Anxiety Inventory (STAI) is one of the most commonly used measures of trait and state anxiety^77^. It consists of 40 items, 20 measuring state anxiety (example: “I am worried”) and 20 measuring trait anxiety (example: “I worry too much over something that really doesn’t matter”).

#### The Beck depression Inventory-II (BDI-II)

The Beck Depression Inventory-II (BDI-II) was used to measure depressive symptoms. The questionnaire contains 21 items, on which the frequency of experiencing different symptoms of depression within the past two weeks are indicated on a Likert-type scale ranging from 0 to 3^78^. One item (Q9 – suicidal ideation) was removed from the questionnaire in in response to local ethics review. The scale has excellent reliability (average Cronbach’s α in a meta-study 0.9) and validity^79^.

#### Sociodemographic data

Sociodemographic factors were also measured, which included age in years, gender (male, female, other), and highest level of education.

### Procedure

The study involved a single online session lasting approximately 20 min. After filling in the demographic questions, participants were administered the State-Trait Anxiety Inventory and the Beck Depression Inventory-II. Then, the participants went through 20 videos with the force choice option on the METV test under a separate link. In each part of the survey, participants were required to share their email address and name. These were used in order to merge the data from the different measures, and then the data was anonymized.

### Data analysis

#### Data screening

Two data screening criteria were used in order to clean the data. First, extreme outliers – defined as those participants whose METV and/or trait STAI test scores lie more than 3 times the interquartile range below the first quartile or above the third quartile – were removed from the dataset. Less extreme outliers – defined as those participants whose METV and/or trait STAI test scores lie between 1.5 and 3 times the interquartile range below the first quartile or above the third quartile – were included in primary analyses but their influence was investigated through sensitivity analyses that exclude them. Participants that did not comply with the basic instructions of the study (e.g., used Chrome browsers or smart phones, which are not compatible with the METV), were removed from the dataset. Furthermore, there were participants who experienced technical difficulties during the study, and their results were not used in the analysis also. Finally, for all statistical analyses conducted for this study, all relevant assumptions (such as normality of data and homoscedasticity of the residuals) were verified prior to analyses.

#### Analysis

Data analysis was conducted in SPSS v25. For the data analyses, we allocated people into two equal groups (low anxiety, high anxiety) based on the trait anxiety sub-scale of the State-Trait Anxiety Inventory (STAI). Similarly, two equal groups were created based on the BDI-II scores (low mood, control). Both separations of participants into groups were done by using the median value. For trait anxiety, the median value was 44. As 55.9% of participants had a score of 44 or less, we have performed all subsequent analyses with both 44 and 43 as the cut-off value, in order to prevent any bias due to more participants being in one of the two groups. There were no differences in results, and the presented results were done with low anxiety group containing participants who scored 44 and less, while the high anxiety group contained participants who scores 45 and more. The median score on BDI-II was 9, with 50.4% of participants scoring 9 or less on BDI-II. Hence, we classified participants scoring 10 or more into the low mood group, while those with scores 9 and below were classified as the control group. This was done in order to create groups as equal in size as possible. Regression analyses were utilized to check for the relationship of trait anxiety (high, low) with the overall METV score, as well as the anger recognition score. A series of Ordinary Least Squares (OLS) linear regression analyses was conducted on the obtained study data. To test H1 and obtain the primary outcome of the study, we compared the METV test scores between the two groups made based on trait anxiety. For H2, the two anxiety groups were compared on the accuracy in detecting angry faces. In the complete models for both hypotheses, the results were adjusted for low mood and the demographic control variables age, sex, and level of education.

## Supplementary Information

Below is the link to the electronic supplementary material.

Supplementary Material 1

## Data Availability

Study data and code used for analysis is available on OSF: https://osf.io/mqud7/.
