# Supplementary material for: Low mood, not anxiety, connected with micro facial expression recognition
Source: Sci Rep. 2025 Nov 28;15:42784. doi: 10.1038/s41598-025-26921-1 (PMC12663378; doi:10.1038/s41598-025-26921-1)
Supplement: Supplementary file 1 — Supplementary Material 1 [file 41598_2025_26921_MOESM1_ESM.docx]

Supplementary materials

**Figure S1** Histogram of scores on trait anxiety subscale of STAI of participants belonging to low and high anxiety groups


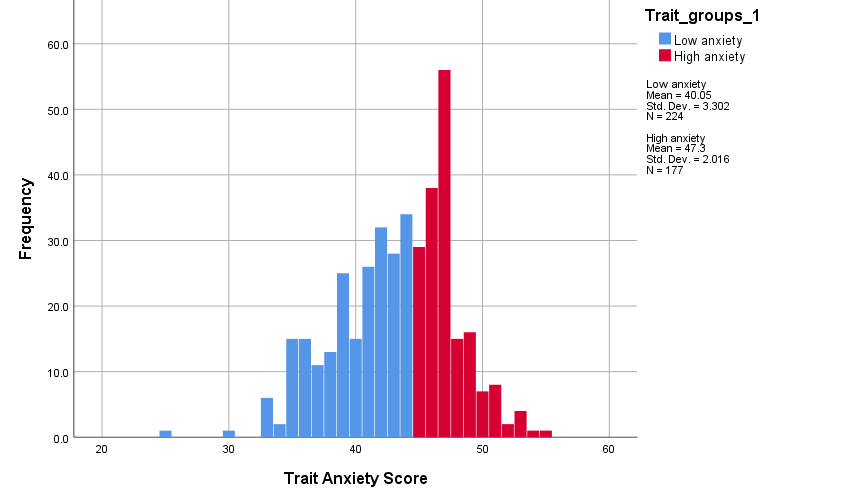


**Table S1** Demographic characteristics of low and high anxiety groups

| Variable | Low anxiety | High anxiety |
| --- | --- | --- |
| Female gender | 46.4 | 54.2 |
| Education level | |  |
| No formal education | 0.4 | 0.6 |
| O-levels | 22.8 | 13.0 |
| A-levels | 23.7 | 23.2 |
| Bachelor’s degree | 37.1 | 44.6 |
| Postgraduate degree | 16.1 | 18.6 |

**Table S2** Descriptive statistics on key study variables of low and high anxiety groups

| Variable | Low anxiety | | | | High anxiety | | | |
| --- | --- | --- | --- | --- | --- | --- | --- | --- |
|  | Mean | Std. dev. | Min | Max | Mean | Std. dev. | Min | Max |
| METV first correct answers | 6.76 | 2.55 | 1 | 14 | 6.66 | 2.51 | 0 | 12 |
| Angry face first correct answers | 0.21 | 0.21 | 0 | 1 | 0.21 | 0.21 | 0 | 1 |
| Trait anxiety | 40.05 | 3.30 | 25 | 44 | 47.30 | 2.02 | 45 | 55 |
| State anxiety | 43.42 | 5.00 | 30 | 58 | 46.86 | 4.08 | 36 | 62 |
| Depression (BDI-II) | 14.10 | 11.01 | 0 | 47 | 11.56 | 13.27 | 0 | 49 |

**Figure S2** Histogram of scores on BDI-II of participants belonging to low mood and control groups
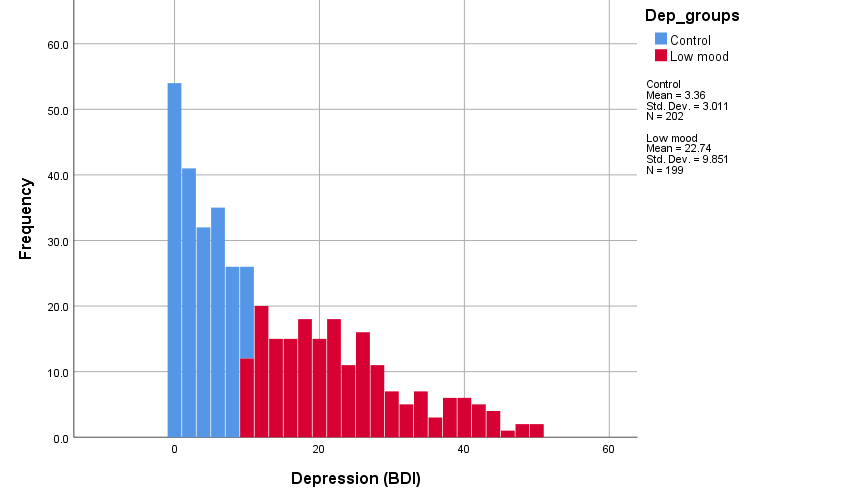


**Table S3** Demographic characteristics of low mood and control groups

| Variable | Low mood (%) | Control (%) |
| --- | --- | --- |
| Female gender | 52.0 | 47.7 |
| Education level | |  |
| No formal education | 1 |  |
| O-levels | 19.6 | 17.3 |
| A-levels | 21.1 | 25.7 |
| Bachelor’s degree | 42.7 | 38.1 |
| Postgraduate degree | 15.6 | 18.8 |

**Table S4** Descriptive statistics on key study variables of low mood and control groups

| Variable | Low mood | | | | Control | | | |
| --- | --- | --- | --- | --- | --- | --- | --- | --- |
|  | Mean | Std. dev. | Min | Max | Mean | Std. dev. | Min | Max |
| METV first correct answers | 7.09 | 2.32 | 1 | 14 | 6.35 | 2.67 | 0 | 14 |
| Sad face first correct answers | 0.40 | 0.26 | 0 | 1 | 0.32 | 0.26 | 0 | 1 |
| Mistaking happiness for neutrality | 0.04 | 0.19 | 0 | 1 | 0.02 | 0.16 | 0 | 1 |
| Mistaking neutrality for sadness | 0.05 | 0.21 | 0 | 1 | 0.02 | 0.14 | 0 | 1 |
| Trait anxiety | 42.56 | 5.20 | 25 | 54 | 43.93 | 3.73 | 33 | 55 |
| State anxiety | 45.99 | 5.38 | 33 | 62 | 43.90 | 4.18 | 30 | 59 |
| Depression (BDI-II) | 22.74 | 9.85 | 10 | 49 | 3.36 | 3.01 | 0 | 9 |

**Table S5** Regression models predicting first attempt METV correct answers based on original STAI trait anxiety and BDI scores

| Predictors | Unadjusted | | Model 1 | | Model 2 | |
| --- | --- | --- | --- | --- | --- | --- |
|  | *b* [95% CI] | *p* | *b* [95% CI] | *p* | *b* [95% CI] | *p* |
| Trait anxiety score | -0.007 | .811 | -0.002 | .929 | -0.001 | .961 |
|  | [-0.061, 0.048] |  | [-0.057, 0.052] |  | [-0.056, 0.053] |  |
| Age |  |  | -0.036 | <.001 | -0.034 | <.001 |
|  |  |  | [-0.052, -0.020] |  | [-0.051, -0.017] |  |
| Gender |  |  | -0.162 | .516 | -0.161 | .519 |
|  |  |  | [-0.652, 0.328] |  | [-0.651, 0.329] |  |
| Education |  |  | 0.032 | .797 | 0.038 | .766 |
|  |  |  | [-0.215, 0.280] |  | [-0.211, 0.286] |  |
| BDI score |  |  |  |  | 0.008 | .48 |
|  |  |  |  |  | [-0.014, 0.029] |  |

**Table S6** Regression models predicting first attempt angry face correct answers based on original STAI trait anxiety and BDI scores

| Predictors | Unadjusted | | Model 1 | | Model 2 | |
| --- | --- | --- | --- | --- | --- | --- |
|  | *b* [95% CI] | *p* | *b* [95% CI] | *p* | *b* [95% CI] | *p* |
| Trait anxiety score | 0.001 | .888 | 0.001 | .886 | -0.001 | .881 |
|  | [-0.004, 0.005] |  | [-0.004, 0.005] |  | [-0.004 0.005] |  |
| Age |  |  | -0.001 | .062 | -0.001 | .083 |
|  |  |  | [-0.003, -0.001] |  | [-0.003, -0.001] |  |
| Gender |  |  | -0.025 | .246 | -0.025 | .247 |
|  |  |  | [-0.066, 0.017] |  | [-0.066, 0.017] |  |
| Education |  |  | 0.001 | .909 | 0.001 | .904 |
|  |  |  | [-0.020, 0.022] |  | [-0.020, 0.022] |  |
| BDI score |  |  |  |  | 0.001 | .895 |
|  |  |  |  |  | [-0.002, 0.002] |  |

**Table S7** Regression models predicting first attempt METV correct answers based on depression

| Predictors | Unadjusted | | Model 1 | | Model 2 | |
| --- | --- | --- | --- | --- | --- | --- |
|  | *b* [95% CI] | *p* | *b* [95% CI] | *p* | *b* [95% CI] | *p* |
| Low mood group | 0.729 | .004 | 0.371 | .166 | 0.368 | .173 |
|  | [0.236, 1.223] |  | [-0.154, 0.897] |  | [-0.162, 0.899] |  |
| Age |  |  | -0.031 | <.001 | -0.031 | <.001 |
|  |  |  | [-0.049, -0.014] |  | [-0.049, -0.014] |  |
| Gender |  |  | -0.145 | .559 | -0.147 | .556 |
|  |  |  | [-0.633, 0.343] |  | [-0.638, 0.344] |  |
| Education |  |  | 0.038 | .763 | 0.039 | .757 |
|  |  |  | [-0.208, 0.283] |  | [-0.209, 0.287] |  |
| High anxiety group |  |  |  |  | -0.025 | .923 |
|  |  |  |  |  | [-0.525, 0.476] |  |

**Table S8** Regression models predicting first attempt sad faces correct answers based on depression

| Predictors | Unadjusted | | Model 1 | | Model 2 | |
| --- | --- | --- | --- | --- | --- | --- |
|  | *b* [95% CI] | *p* | *b* [95% CI] | *p* | *b* [95% CI] | *p* |
| Low mood group | 0.087 | <.001 | 0.073 | .009 | 0.074 | .009 |
|  | [0.037, 0.138] |  | [0.019, 0.128] |  | [0.018, 0.129] |  |
| Age |  |  | -0.001 | .208 | -0.001 | .208 |
|  |  |  | [-0.003, 0.001] |  | [-0.003, 0.001] |  |
| Gender |  |  | -0.029 | .262 | -0.029 | .267 |
|  |  |  | [-0.080, 0.022] |  | [-0.080, 0.022] |  |
| Education |  |  | 0.005 | .698 | 0.002 | .936 |
|  |  |  | [-0.020, 0.031] |  | [-0.050, 0.054] |  |
| High trait anxiety group |  |  |  |  | 0.002 | .936 |
|  |  |  |  |  | [-0.050, 0.054] |  |

**Table S9** Regression models predicting mistaking happy faces for neutral based on depression

| Predictors | Unadjusted | | Model 1 | | Model 2 | |
| --- | --- | --- | --- | --- | --- | --- |
|  | Exp*(B)* [95% CI] | *p* | Exp*(B)* [95% CI] | *p* | Exp*(B)* [95% CI] | *p* |
| Low mood group | 0.603 | .382 | 0.514 | .514 | 0.484 | .247 |
|  | [0.194, 1.875] |  | [0.153, 1.729] |  | [0.142, 1.654] |  |
| Age |  |  | 1.013 | .504 | 1.013 | .529 |
|  |  |  | [0.974, 1.054] |  | [0.974, 1.053] |  |
| Gender |  |  | 0.820 | .727 | 0.812 | .714 |
|  |  |  | [0.269, 2.497] |  | [0.266, 2.475] |  |
| Education |  |  | 1.169 | .592 | 1.153 | .629 |
|  |  |  | [0.660, 2.071] |  | [0.647, 2.053] |  |
| High trait anxiety group |  |  |  |  | 0.461 | .461 |
|  |  |  |  |  | [0.204, 2.056] |  |

**Table S10** Regression models predicting mistaking neutral faces for sad based on depression

| Predictors | Unadjusted | | Model 1 | | Model 2 | |
| --- | --- | --- | --- | --- | --- | --- |
|  | Exp*(B)* [95% CI] | *p* | Exp*(B)* [95% CI] | *p* | Exp*(B)* [95% CI] | *p* |
| Low mood group | 0.424 | .159 | 0.297 | .063 | 0.291 | .062 |
|  | [0.128, 1.401] |  | [0.083, 1.066] |  | [0.080, 1.062] |  |
| Age |  |  | 1.032 | .110 | 1.032 | .112 |
|  |  |  | [0.993, 1.074] |  | [0.993, 1.073] |  |
| Gender |  |  | 1.109 | .857 | 1.108 | .857 |
|  |  |  | [0.361, 3.405] |  | [0.361, 3.403] |  |
| Education |  |  | 1.246 | .459 | 1.244 | .464 |
|  |  |  | [0.696, 2.230] |  | [0.694, 2.229] |  |
| High trait anxiety group |  |  |  |  | 0.892 | .844 |
|  |  |  |  |  | [0.286, 2.782] |  |
